# Where do GPs find patients with possible palliative care needs? A cross-sectional descriptive study

**DOI:** 10.3399/BJGPO.2020.0100

**Published:** 2021-03-03

**Authors:** Bert Leysen, Bart Van den Eynden, Johan Wens

**Affiliations:** 1 Primary and Interdisciplinary Care, Palliative Care Research Group, University of Antwerp, Antwerp, Belgium; 2 Multidisciplinary Pain Centre, Palliative Care Support Team, University Hospital of Antwerp, Antwerp, Belgium

**Keywords:** epidemiology, terminal illness, palliative care, quality assurance, general practice

## Abstract

**Background:**

For GPs to implement early palliative care, the first step is to identify patients with palliative care needs. The surprise question (SQ) is a screening tool that aims to aid this identification; for example, a response of 'no' to the SQ — 'Would you be surprised if this patient would die within a year?' — would suggest palliative care may be needed.

**Aim:**

To describe setting-specific screening results of patients eligible for early palliative care in family practices, which is defined as patients aged **≥**45 years with GPs' responses of 'no' to the SQ.

**Design & setting:**

A secondary analysis was undertaken using a cross-sectional descriptive study in family practices in five areas in Belgium.

**Method:**

GPs were recruited by targeted sampling. As a first part of an implementation research project, participating GPs provided demographic information about themselves and also provided a response to the SQ for all patients who came to the practice in 10 consecutive office days. A summary table describing the sex, age, location of contact (GP surgeries, patients' homes, or nursing homes) of the patients was provided by each GP.

**Results:**

Fifty-six GPs provided complete data for the practice summary tables. In total, 9150 patients were described (all ages, all settings), of which 506 patients (6%) had a GP response of 'no' to the SQ. The distribution of SQ-no-as-answer patients per setting was: 152/7659 (2%) patients seen in family practice surgeries; 139/998 (14%) patients seen in their homes; and 215/493 (44%) patients seen in nursing homes.

**Conclusion:**

There was a large number of patients with SQ-no-as-answer, with possible palliative care needs. To enhance implementation of early palliative care, future research should compare results of SQ and other screening tools with palliative care symptoms assessments.

## How this fits in

Primary care practitioners should screen for possible palliative care needs. The SQ is a method for palliative care needs screening. In 10 consecutive working days 56 GPs registered 6% no answers to the SQ in a population of 9150 patients, which is considered a large proportion of this group. Therefore, this cross-sectional study adds to the evidence that palliative care needs are prevalent in primary care.

## Introduction

Offering palliative care to patients should be based on palliative care needs rather than on a terminal prognosis. This is considered a task for all physicians, and particularly for GPs because they are in close contact with the largest part of the population.^1^

In many countries, the SQ,^2^ alone or in combination with other prognostic tools, is suggested as a screening method for palliative care needs.^1,3^ A patient for whom a 'no' answer to the SQ is given (for example, 'No, I would not be surprised if this patient would die within a year'), is expected to have palliative care needs.^4,5^

Pro-Spinoza was a Belgian project, sponsored by the National Institute for Health and Disability Insurance, which evaluated the implementation of the care pathway for primary palliative care.^6–8^ In this project, there were two eligibility criteria for early palliative care: being aged ≥45 years; and the GP stating a no response to the SQ about the patient.^8^

In Belgium, family practice is known to be transitioning from mostly single-handed practices towards more group practices.^9^ Patients are free to choose their GP anytime they want to see a GP. That is why there are no patient lists available for individual family practices. The lack of clearly defined practice populations complicates calculations of practice prevalences.^10,11^

This secondary study within the larger pro-Spinoza study had two aims, both contributing to the understanding of implementing the complex intervention of screening of (early) patients who may need palliative care.^12^ The first aim was to report SQ screening results in Belgian family practices, according to setting (location of contact), which could be: GP surgeries, patients’ homes, and nursing homes. The second aim was to compare the characteristics of participating GPs who completed data collection on the SQ with participating GPs who did not complete data collection.

## Method

### Study design

This was a cross-sectional study of GP practices in Belgium and is reported following the STROBE-checklist^13^ (see Supplementary Table S1).

For this study the researchers decided to use a pragmatic method, that is, by asking GPs to register a list of the patients seen in 10 consecutive working days. This means that this study is not a population-based survey. It is a cross-sectional study of patients coming to the participating GP practices (that is, always for a clinical reason).

### Setting

This project ran in five areas in Belgium (Antwerp, Brussels, Hasselt, Mons, and Namur). These areas encompass five palliative care networks.^8^ Recruitment of participating GPs occurred from January 2014 until March 2016.

### Participants

Recruitment methods included: educational sessions, GP practice visits, letters, emails, and phone calls.^14^ Approximately every 6 months, another area started recruitment and data collection.^8^ This means that some areas had more recruitment and data collection time than others.

The recruitment of local GPs was facilitated as much as possible by already existing relationships of local GPs with the research team, with the palliative care networks, or with the GPs’ circles. GPs’ circles are local associations of mostly 50–100 GPs, with a main purpose of organising out-of-hours duties and postgraduate education for their members. The only exclusion of GPs was the refusal to participate. The research team recruited a diverse group of GPs (for example, single-handed versus group, fee-for-service versus capitation based, male versus female, and diverse age groups). More details about recruitment methods are described elsewhere.^14^

### Sample size

The study had a targeted sample of GPs. The sample size calculated for the main study was 180 GPs.^8^ A formal sample size calculation has not been done for this secondary analysis, which serves as an exploration of this issue.

### Variables

As part of the pro-Spinoza study,^8^ participating GPs were asked to record prospectively the following information about all patients seen in 10 consecutive working days: sex, age, location of contact (in the GP surgery, at a patient’s home during a home visit, or in a nursing home), and their 'yes' or 'no' answer to the SQ ('Would you be surprised if the patient died within a year?'). Through a secure, online platform, GPs provided demographic information about themselves in addition to the two standardised summary tables that provided the responses about the patients (see Supplementary Box S1 for the questionnaire).

The summary table about SQ prevalence focused on patients aged ≥45 years. This choice was made based on two reasons: 1) regarding follow-up, dying people aged <45 years are (at least in Belgium) expected to be followed up more closely by specialists than by GPs. Starting from age 45 years, dying people are expected to be followed up at least partly by family doctors; and 2) the authors did not want to restrict the research population to the older, retired population, because that would limit the diversity of palliative care pathologies.

### Statistical methods

The data from the GPs were checked by the researchers. Any summary tables that were incomplete, contained the same number values throughout, or included guesses (for example, sex distribution was exactly 75%/25%) were removed from the analysis. The analysis was a description in percentages of proportions found: general demographics of the full sample, and proportions of patients with SQ-no-as-answer per setting and per age or sex category.

Because one of the main goals of the larger project was to obtain insights in implementation mechanisms, a simple comparison was made between participating GPs having filled in the summary tables correctly and those who filled in the tables incorrectly for the relevant GPs’ background characteristics. IBM SPSS Statistics (version 25) was used.

## Results

### Participant characteristics

Of 112 participating GPs, 65 filled in a baseline questionnaire. Of these, nine summary tables were excluded owing to incomplete data. Therefore, 56 GP practices were included in the final analysis. Table 1 provides the comparison between those included and those excluded. Supplementary Table S2 provides the original dataset, and shows why nine data rows were excluded.

**Table 1.** Participant characteristics compared between GPs with complete summary tables and GPs with incomplete summary tables

	**Complete tables, *n* (%)**	**Incomplete tables, *n* (%)**
**Total**	56 (100)	9 (100)
**Sex**		
Male	26 (46)	7 (78)
Female	30 (54)	2 (22)
**Experience as GP, years**		
<10	23 (41)	3 (33)
10–30	20 (36)	1 (11)
>30	13 (23)	5 (56)
**Self-declared time schedule**		
Full-time GP	40 (71)	8 (89)
Part-time GP	16 (29)	1 (11)
**Extra activities, *n***		
0	21 (38)	3 (33)
1	23 (41)	4 (44)
≥2	12 (21)	2 (22)
**Patients in palliative care in the past year, *n***		
0	6 (11)	1 (11)
1–5	36 (64)	4 (44)
6–10	11 (20)	3 (33)
>10	3 (5)	1 (11)
**Type of practice**		
Single-handed	11 (20)	4 (44)
Duo	9 (16)	1 (11)
Group	31 (55)	2 (22)
Community health centre	5 (9)	2 (22)
**Administrative support**		
Administrative support	33 (59)	5 (56)
No administrative support	23 (41)	4 (44)
**Paramedical support**		
Paramedical support	26 (46)	5 (56)
No paramedical support	30 (54)	4 (44)

Although not significant, there were some remarkable differences between the two groups in Table 1. Particularly males, GPs in their early or in their late career phase, GPs with >5 patients in palliative care in the past year, and single-handed GPs provided incomplete data for the practice population registration tables.

### Proportion of patients eligible for early palliative care

The GPs registered 9150 patients in the 10 consecutive working days window (that is, an average of 16 patients on a working day), of which 5479 patients (60%) were aged **≥**45 years, 5462 patients (60%) were female and 7659 patients (84%) were seen in GP surgeries (Table 2). Five hundred and six patients (6%) were aged **≥**45 years and also had a 'no' response to the SQ. This means that approximately one in 20 patients of all ages (*n* = 506/9150), or one in 11 patients aged ≥45 years (*n* = 506/5479), who were seen by a GP were possibly eligible for (early) palliative care (Table 3).

**Table 2.** General demographic characteristics of described patients

	**Male, *n***(**% of general total**)	**Female, *n***(**% of general total**)	**Total, *n***(**% of general total**)
**Aged <45** **years**	1516 (17)	2155 (24)	3671 (40)
**Aged** **≥** **45 years**	2172 (24)	3307 (36)	5479 (60)
**Total**	3688 (40)	5462 (60)	9150 (100)

**Table 3.** Distribution of patients eligible for early palliative care, per setting

	**Total, *n***	**Aged ≥45 years AND a response of 'no' to the SQ, *n***	**%**
**GP surgery**	7659	152	2
**Patients' home**	998	139	14
**Nursing home**	493	215	44
**Total**	9150	506	6

SQ = surprise question.

Table 2 shows the general demographics of this total patient population. Of 9150 patients, 5479 patients (60%) were aged ≥45 years and 5462 patients (60%) were female.

Table 3 shows the total number of patients per setting (GP surgeries, patients' home visits, or nursing home visits) and the number of patients who were eligible for early palliative care; that is, being aged ≥45 years and having a response of 'no' to the SQ. The prevalence of eligible patients was 2% in GP surgeries, 14% in patients’ homes, 44% in nursing homes, and 6% overall.

All data of the summary tables are to be found in Supplementary Table S3.

## Discussion

### Summary

The primary aim of this secondary analysis was to report SQ screening results in Belgian family practices. GPs registered 9150 patients over 10 consecutive working days and answered the SQ for all patients aged **≥**45 years. This study found that one in 11 patients aged ≥45 years (*n* = 506/5479), and approximately one in 20 patients of all ages (*n* = 506/9150) were considered eligible for early palliative care and should be screened more thoroughly for palliative care needs; for instance, by a palliative care symptoms assessment like the (Integrated) Palliative Outcome Scale.^15,16^

In the setting of home visits, the proportion of early palliative care eligibility rose to approximately one in ten patients. In the setting of visits to nursing homes, where all residents are aged **≥**45 years, this prevalence rose to almost one in two patients.

Characteristics related the most (although not significantly) with GPs not having completed data collection were: male sex, late career phase, and single-handed practice. Lack of significance could be related to the small sample size.

### Strengths and limitations

This is one of the first Belgian studies reporting screening results of patients with a need for early palliative care in primary care, which is operationalised as patients with SQ-no-as-answer aged ≥45 years. However, other studies comprise the general population^5,17^ and/or focus on people aged ≥65 years,^5^ or ≥75 years,^18^ in this study the focus was on the age ≥45 years. Focusing on the same age group as in other studies could have improved the comparability with these other studies.

The assessment of patients with the SQ has shown, in this study, to be a feasible intervention. It could be a possible important first step in identifying patients who might benefit from anticipatory care planning and palliative care.

In the parent study, there is a risk for selection bias on the level of the GP practices. These participating GPs are perhaps not representative of GPs in Belgium. A Belgian GP workforce capacity study^19^ showed that in 2016, 59% of active GPs were male (while 46% of 56 GP responders in the present study were male, see Table 1) and that 52% of active GPs were aged >55 years (while only 23% of 56 GP responders in the present study practised medicine >30 years, see Table 1).

Furthermore, there might be a selection bias of patients: only patients visiting their GPs with clinical problems were included; thus, raising the risk of palliative care needs compared with the whole patient population. Nevertheless, selecting only patients a GP had contact with reflects daily GP practice, and narrows the research–practice gap.

Recruitment and data collection support strategies are described elsewhere in more detail.^14^ Many GPs stated that the online data collection was difficult to handle.^14^ This methodological challenge reduced the available amount of data.

### Comparison with existing literature

A Belgian study investigated the use of the Palliative Care Indicators Tool (PICT), a modified Supportive and Palliative Care Indicator Tool (SPICT),^20^ including the SQ, followed by general and disease-specific indicators similar to the SPICT indicators.^17^ In the report, there were no separate data available on the prevalence of patients with SQ-no-as-answer, but 4% of patients seen by participating GPs were considered PICT positive and 14% of patients in nursing homes were considered PICT positive.^17^ This is a lower number than in patients in nursing homes in the present study (44%), potentially because of the extra criteria of the PICT on top of the SQ.

In a Catalonian study, the SQ-no-as-answer prevalence was 1% in the primary care setting (of one primary care centre) and 62% in the nursing home.^5^ The NECPAL ('Necesidades Paliativas') positive prevalence (answer was no to the SQ and positive in one of the NECPAL domains) was similar in this study: 1% in the primary care setting and 54% in the nursing homes.^5^ Here, the general SQ-no-as-answer prevalence is lower than in the present study, because their study investigated the whole practice population and the present study only investigated patients seen during 10 days. The nursing homes in Catalonia have a higher percentage of patients with SQ-no-as-answer (62%) than in the present study (44%). This difference could be based on differing nursing home demographics. In Belgium, there are around 70 beds in residential long-term care facilities per 1000 people aged >65 years, while in Spain (for Catalonia alone, no data were found) there are around 45 similar beds per 1000 people of this age group.^21^

An Australian randomised controlled trial compared the predictive value of screening tools (SQ or SQ followed by SPICT) versus unguided intuition.^22^ GPs identified more patients at risk of dying using SQ (11.8%) than when they used intuition (5.4%, *P* = 0.01). Screening tools had higher sensitivity and lower specificity than intuition, but there was no difference in positive or negative predictive value.

In a Dutch case vignette study,^23^ the original SQ (SQ1: 'Would I be surprised if this patient were to die within a year?') is considered to have a low specificity, leading to overidentification of patients eligible for early palliative care. To make early palliative care more feasible, a second SQ was added (SQ2: 'Would I be surprised if this patient is still alive after 12 months?'). These two SQs together encouraged GPs to deliver anticipatory palliative care, compared with the original SQ alone, and compared with having no SQ. In a Dutch explorative study,^18^ two GPs answered both SQs for their patients aged ≥75 years. Of 292 patients, SQ1 was answered with 'no' for 161 patients. Of these, SQ2 was answered with 'yes' in 22 patients. This group (SQ1-no-answers, SQ2-yes-answers) discussed more frequently and more palliative care aspects with the GP. So, the ‘double surprise question’ seems a useful enhancement of the original SQ.

### Implications for research and practice

The most important implication from this research is that palliative care needs are prevalent in primary care. Approximately one in 20 patients of all ages, seen in 10 consecutive working days, possibly have palliative care needs. To recognise these needs will remain the first step of any care pathway or protocol. However, to study whether the SQ or any other screening tool is a valid proxy for palliative care needs, a prospective study is recommended in which results of screening tools are compared with palliative care needs and symptoms assessments, such as the Integrated Palliative Outcome Scale.^17^

The methodology adopted in the parent study of this secondary analysis shows that it is feasible to obtain responses online. However, this led to data being excluded owing to it being incomplete.

GPs who were male, single-handed, and/or in their late career phase, seemed less involved in primary palliative care research activities than other GP profiles, possibly because they lacked support with data collection.^14^ These preliminary findings could refine recruitment and participant monitoring strategies in primary palliative care research.

This research suggests that GPs visiting patients at home or in a nursing home should be mindful of palliative care needs owing to the higher context-specific proportions in these locations.

In conclusion, the SQ appears a feasible screening tool for GPs. When asking data collection from GPs, a research team should offer the most support to male GPs who work single-handed and who are in their last career phase.

The proportion of patients with SQ-no-as-answer is high in primary care settings, particularly in nursing homes. A 'no' answer to the SQ is not enough to warrant high-intensity palliative care to a specific patient, but should be a trigger to think of ways to integrate anticipatory care planning and palliative care needs assessments in the daily care of the patient.
